# Exploring the impact of encapsulation on the stability and bioactivity of peptides extracted from botanical sources: trends and opportunities

**DOI:** 10.3389/fchem.2024.1423500

**Published:** 2024-07-10

**Authors:** Viridiana Pérez-Pérez, Cristian Jiménez-Martínez, Jorge Luis González-Escobar, Luis Jorge Corzo-Ríos

**Affiliations:** ^1^ Departamento de Bioprocesos, Unidad Profesional Interdisciplinaria de Biotecnología, Instituto Politécnico Nacional (IPN), México City, Mexico; ^2^ Departamento de Ingeniería Bioquímica, Escuela Nacional de Ciencias Biológicas, Instituto Politécnico Nacional (IPN), Mexico City, Mexico; ^3^ Instituto Tecnológico de Ciudad Valles, Tecnológico Nacional de México, Ciudad Valles, San Luis Potosí, Mexico

**Keywords:** plant protein, bioactive peptide, encapsulation, wall materials, controlled delivery

## Abstract

Bioactive peptides derived from plant sources have gained significant attention for their potential use in preventing and treating chronic degenerative diseases. However, the efficacy of these peptides depends on their bioaccessibility, bioavailability, and stability. Encapsulation is a promising strategy for improving the therapeutic use of these compounds. It enhances their stability, prolongs their shelf life, protects them from degradation during digestion, and enables better release control by improving their bioaccessibility and bioavailability. This review aims to analyze the impact of various factors related to peptide encapsulation on their stability and release to enhance their biological activity. To achieve this, it is necessary to determine the composition and physicochemical properties of the capsule, which are influenced by the wall materials, encapsulation technique, and operating conditions. Furthermore, for peptide encapsulation, their charge, size, and hydrophobicity must be considered. Recent research has focused on the advancement of novel encapsulation methodologies that permit the formation of uniform capsules in terms of size and shape. In addition, it explores novel wall materials, including polysaccharides derived from unconventional sources, that allow the precise regulation of the rate at which peptides are released into the intestine.

## 1 Introduction

Scientific evidence has shown a relationship between food consumption and human health. Protein is one of the food components most strongly linked to health because it is necessary for the development and function of cells, tissues, organs, and systems. The nutritional value of protein depends on its amino acid composition and bioavailability (Karami and Akbari-Adergani, 2019; Montesano et al., 2020). High-quality proteins must contain all the essential amino acids required for muscle and organ protein synthesis. One classification of dietary proteins divides them based on their source into animal and plant proteins. Animal proteins typically have a higher biological value than vegetable proteins due to their adequate balance of essential amino acids. However, accurate plant proteins may have a distinctive essential amino acid composition (Montesano et al., 2020).

There is a relationship between the consumption of some plant proteins and health benefits such as reduced blood pressure and cholesterol levels, as well as anti-inflammatory, anti-cancer, and immunomodulatory activities (Samtiya et al., 2021; Ali et al., 2022). The peptides obtained from the hydrolysis of these proteins, known as bioactive peptides, are mainly responsible for these effects. Protein hydrolysis occurs naturally in the human digestive process. However, in recent years, alternative means, such as enzymatic, physical, chemical, and biological methods, have been used to generate peptides for specific purposes (Juárez-Chairez et al., 2022).

Bioactive peptides are small fragments, generally from 2 to 50 units of amino acids linked by peptide bonds (Manzanares et al., 2019; Fan et al., 2022). The biological activity of peptides is contingent upon their amino acid sequence, which is influenced by the protein source and the hydrolysis method employed. The biological activity of peptides is greater than that of the amino acids free because the physicochemical properties of the peptides depend on the amino acid sequence. (Gallegos-Tintoré et al., 2013). Therefore, to preserve the biological activity of these compounds, it is essential to avoid their hydrolysis during food processing, food storage, and during their passage through the gastrointestinal tract. (Juárez-Chairez et al., 2022).

Bioactive peptides derived from plant sources can reduce levels of hypertension and type 2 diabetes mellitus because these peptides can inhibit the activity of angiotensin-converting enzyme (ACE) and dipeptidyl peptidase IV (DPP-IV), whose activity is closely related to the development of hypertension and blood glucose accumulation (Cian et al., 2019; Li et al., 2019; Pugliese et al., 2019; Suárez and Añón, 2019; Ying et al., 2021). Some studies indicate that peptides with a sequence of between 2 and 8 amino acids containing hydrophobic residues such as aspartic acid, glutamic acid, and proline can inhibit the activity of these enzymes (Sato et al., 2018). Other bioactivities associated with peptides derived from plant sources are antioxidant and anticancer properties (Mazloomi et al., 2020; Ilhan-Ayisigi et al., 2021). Peptides with antioxidant activity usually contain amino acids such as proline, histidine, tyrosine, tryptophan, methionine, and cysteine in their sequences and hydrophobic amino acids such as valine and leucine at the end of the chain (Gallegos-Tintoré et al., 2013).

Bioactive peptides derived from plant sources have low physicochemical stability and can be hydrolyzed before reaching the target organs, so their application in functional foods, supplements, or drug formulation is limited (Velarde-Salcedo et al., 2013; Karami and Akbari-Adergani, 2019).Therefore, encapsulation is an alternative to preserve the bioactivity of peptides (Li et al., 2023). However, the size, amino acid sequence, and solubility of peptides must be considered in the encapsulation method and wall material selection. Since most bioactive peptides derived from plant sources contain hydrophobic amino acids, wall materials and encapsulation techniques that can stabilize them should be selected.

**FIGURE 1:**
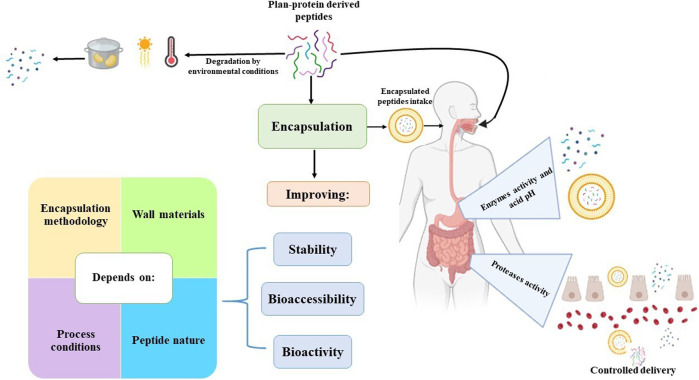
An overview of plan-protein-derived peptides encapsulation.

The impact of encapsulation on the bioactivity and stability of peptides derived from plant sources is contingent upon the encapsulation methodology, the wall materials utilized, and the conditions employed during the encapsulation process. These variables dictate the shape, size, structure, and physicochemical properties of the capsules, which are closely associated with peptide stability during storage and the controlled release of peptides (Figure 1).

Due to the biological activity of peptides derived from plant sources, the potential of new encapsulation techniques, in addition to the use of mucilage and other unconventional gums as wall materials, has recently been explored. These technologies may represent a promising way to maintain the integrity and biological activity of peptides, however, it is important to study the effect of the new techniques developed on the stability and controlled release of bioactive peptides.

Therefore, this review presents the most commonly used encapsulation techniques and wall materials, as well as a set of emerging trends and promising opportunities for improving the encapsulation efficiency of plant-derived bioactive peptides and analyzes the impact of encapsulation techniques and wall materials on their bioactivity and controlled release.

## 2 Influence of the digestion process and stability of bioactive peptides on biological effects

**FIGURE 2:**
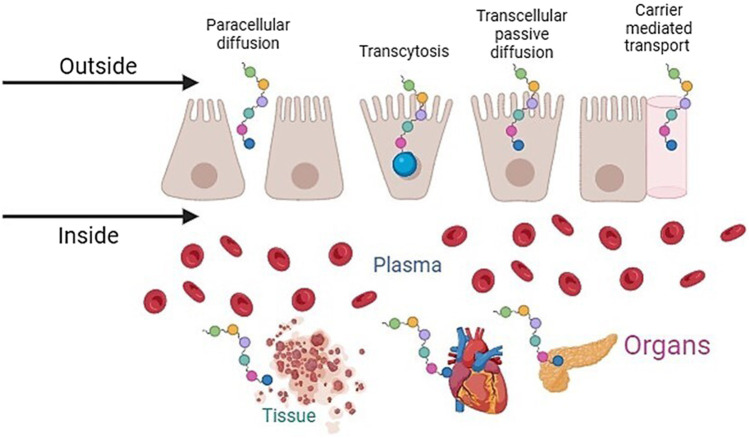
Plan-protein-derived peptides uptake mechanisms.

The absorption of bioactive peptides has a significant influence on their biological effects. During the digestion process, bioactive peptides must resist the activity of enzymes present in the gastrointestinal tract to reach the target organs in adequate concentrations without being hydrolyzed so that they can exert their biological effect (Karaś, 2019; Bhandari et al., 2020). The hydrolysis of peptides before absorption diminishes their bioactivity. In the small intestine, many proteases are distributed between the lumen and in the membrane at the brush border of the epithelial cells, which can hydrolyze them before they are absorbed. Peptides are absorbed in the small intestine, specifically in the jejunum, by the following mechanisms: paracellular diffusion, transcellular passive diffusion, transcytosis, and carrier-mediated transport, as shown in Figure 2 (Xu et al., 2019; Jakubczyk et al., 2020).

In paracellular diffusion, peptides pass through water-filled pores between intestinal epithelial cells. Transcellular passive diffusion allows peptides to pass down a concentration gradient. This mechanism depends on the peptide size, charge, and hydrophobicity (Wu et al., 2024). On the other hand, transcytosis is a mechanism that requires energy to transport peptides from one side of the polarized cell to the other. This mechanism favors the transport of long-chain and hydrophobic peptides (Komin et al., 2017). Carrier-mediated transport involves the movement of peptides against a concentration gradient via specific transporters located in the cell membrane. The transporters allow dipeptides and tripeptides to be absorbed intact across the intestinal membrane into the bloodstream and prevent them from being hydrolyzed by intracellular peptidases present in enterocytes. One of the transmembrane peptide transporters is PepT1 (Okagu et al., 2021).

In PepT1 carrier-mediated permeation, absorption depends on the electrochemical proton gradient between the intestinal lumen and the epithelial cells, as well as on the affinity of the peptides for the carrier. This mechanism absorbs small peptides because the size of the cavity through which they pass is 13 × 12 × 11 Å. The binding cavity has 12 transmembrane regions that, together with the hydrophobic region, are prominent in the binding of the H^+^/peptide complex and the uptake of the peptides by enterocytes (Shen and Matsui, 2019; Xu et al., 2019). Once in the bloodstream, most absorbed peptides are hydrolyzed by peptidases in the blood, so they do not reach the target organs, considerably reducing their biological activity (Daliri et al., 2017).

Another factor affecting the biological activity of bioactive peptides is the changes that occur during food processing and storage. Some physical factors used in food processing, such as light, temperature, and radiation, may modify the structure of proteins and peptides, decreasing their biological activity (Toldrá et al., 2018; Bhandari et al., 2020). In addition, molecular interactions of peptides with other components of the food matrix, such as reducing sugars, modify their structure and function. Therefore, it is important to determine the conditions under which peptides should be processed and stored to preserve their biological activity (Daliri et al., 2017). Thus, encapsulation of bioactive peptides is a helpful tool to preserve their biological activity.

## 3 Encapsulation techniques

Encapsulation is one of the most widely used methods for preserving the functional and physicochemical properties of bioactive compounds. It has allowed the incorporation of various compounds (natural ingredients, volatile compounds, bioactive compounds, enzymes, etc.) into complex food matrices. Encapsulation is a process by which solid or liquid particles are surrounded by a coating or embedded in a matrix (Jamekhorshid et al., 2014; Bakry et al., 2016). The outcome achieved through the encapsulation process comprises capsules, particles, or spheres. These entities are classified according to their size into nanocapsules (less than 1 µm), microcapsules (between 3 and 800 µm), or macrocapsules (larger than 1,000 µm) (Jyothi et al., 2010; Sobel et al., 2014).

**FIGURE 3:**
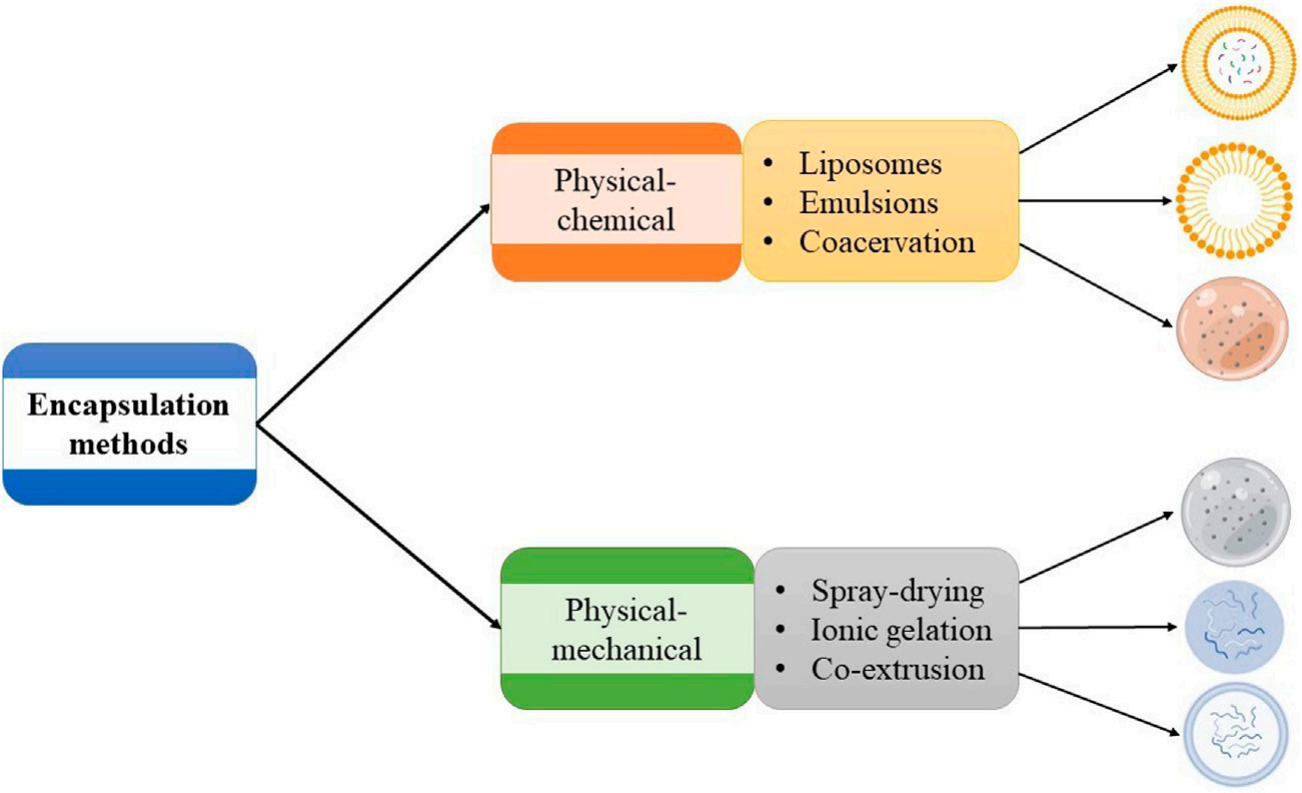
Classification of plan-protein-derived peptides encapsulation methods.

The primary functions of encapsulation are to shield the active compound from decay, release it under specific conditions at regulated rates, enhance its flow properties, and preserve its sensory characteristics (Nazzaro et al., 2012; Sobel et al., 2014). The encapsulation efficiency of bioactive compounds depends on the wall material and encapsulation method employed (Ramakrishnan et al., 2018). Some approaches used in peptide encapsulation are categorized in Figure 3 as physical-chemical and physical-mechanical. The selection of an encapsulation technique depends on the type of bioactive compound and wall material used and the preferred encapsulation traits. The following are the main methods used to encapsulate bioactive peptides.

### 3.1 Spray drying

Spray-drying microencapsulation involves dissolving or dispersing a bioactive compound in a solution of wall material, ultimately resulting in a solution, emulsion, or suspension. The spray drying process is a unitary operation in which a liquid product is fed and atomized in a hot convective medium (usually air). It comprises four stages. The first stage consists of the formation of little droplets during the atomization of the feed. The second stage is when the droplets come into contact with the hot air, as a result of which the third stage begins, which consists of rapid evaporation of the solvent, forming a thin film of the wall material, converting the liquid feed into fine solid particles, which are recovered in the fourth stage of the process (Fang and Bhandari, 2010; Plazola-Jacinto et al., 2019).

The loss of moisture from the particles formed is divided into two stages: one of constant and another of decreasing velocity. In the first case, the droplet temperature increases, causing water migration from the interior to the surface particle (Keshani et al., 2015). This moisture migration keeps the droplet surface saturated. Therefore, the resistance in the gas phase controls moisture loss. The partial vapor pressure in the boundary layer surrounding the droplet remains constant, so the evaporation rate per unit surface area is constant. During this stage, the concentration of solutes in the droplet increases, while its size decreases (Shishir and Chen, 2017). The droplet surface becomes dry during a decreasing velocity period, creating a moisture concentration gradient within the droplet. As a result, the drying rate is significantly influenced by the transfer of moisture within the droplet. As water is lost at the surface, the temperature of the droplet rises, and its moisture content falls until it reaches a dry state (Ishwarya et al., 2015).

The microcapsules are collected at the bottom of the dryer, where the heavier particles are, and then separated from the moist air by a cyclone outside the dryer, which isolates the finer particles. The hot air entering the system reaches a temperature between 100°C and 200°C. However, the microcapsules are prevented from reaching temperatures above 60°C due to their short drying time, which protects the product from degradation (Keshani et al., 2015; Samantha et al., 2015). Several technological factors must be considered when spray drying. These include the inlet and outlet temperature of the drying air, the product feed flow rate, the residence time, and the composition of both the wall material and the bioactive compound. Nonetheless, the wall material composition greatly influences the stability of the resulting microcapsules, so the materials used should have high water solubility, low viscosity, and emulsifying ability (Nandiyanto and Okuyama, 2011; McClements, 2018).

Spray drying is a cost-effective and highly flexible process that operates continuously and under automatic control. It is suitable for heat-sensitive materials and has the added advantage of allowing the use of various wall materials (Sarabandi et al., 2020). It is one of the techniques employed in bioactive peptides derived from plant source encapsulation (flaxseed, rapeseed, and bean), the efficiency of this encapsulation technique improves with an increasing amount of wall material since this increase allows several intermolecular interactions to take place between the peptides and the wall material (hydrogen bonds between the C-O groups of the peptides and the O-H groups of the wall material), which stabilizes the peptide-encapsulated material. As a consequence of the increased solids in solution, the encapsulation yield also increases (Wang et al., 2015; Akbarbaglu et al., 2019).

Characteristics of capsules depend on the operating parameters, type, and the proportion of the wall material employed. As the quantity of wall material increases, the size of the resultant capsules tend to grow because of the rise in the viscosity of the feed solution, which gives rise to the generation of larger droplets during atomization (Wang et al., 2015; Akbarbaglu et al., 2019). Furthermore, droplets of varying sizes are produced, which facilitates the formation of agglomerates and increases the particle size distribution (Wang et al., 2015). An increase in size and particle size distribution may present a disadvantage in the controlled release of peptides. Therefore, it is necessary to employ wall materials that permit obtaining capsules with uniform sizes and high yield and encapsulation efficiency values.

The moisture content and water activity of the peptide capsules obtained by spray drying are low, allow for the absence of biological activity degradation during storage, which increases shelf life and preserves the biological properties of bioactive peptides (antioxidant activity, hypoglycemic activity, ACE-inhibitory activity) encapsulated enhancing their potential use in the design of functional foods (Wang and Selomulya, 2020; Berraquero-García et al., 2023).

### 3.2 Ionic gelation

One of the physicochemical encapsulation modalities is ionic gelation. This approach has become increasingly popular in recent years owing to its novelty and appeal and does not use organic solvents or high temperatures (Arenas-Jal et al., 2020). This method is based on ionic interactions between two oppositely charged compounds, either two polymers or a polymer and a divalent ion. The encapsulation process begins by forming an aqueous polymer solution, which interacts with an oppositely charged compound, reacting and forming an insoluble gel. Typically, the active substance is incorporated into the polymer solution, resulting in a solution, a dispersion, or an emulsion (Demont et al., 2016; Kurozawa and Hubinger, 2017; Cano-Sampedro et al., 2020). Gel formation occurs during the atomization of the solution, dispersion, or emulsion into a solution of divalent ions under constant agitation. The droplets that come into contact with the ionic solution generate spherical gel structures that encapsulate the active ingredient evenly distributed across the polymer matrix. The process takes place in two stages: capsule formation and hardening. The first phase determines the dimensions of the particles, while the second phase facilitates interactions between the wall materials and the divalent ions, allowing the capsule to solidify (Ching et al., 2015).

The encapsulation efficiency of encapsulated peptides by ionic gelation depends on the interactions between wall materials and divalent ions. Divalent ions must be available to interact with the polymers to form the network in which the peptides are trapped (Ilhan-Ayisigi et al., 2021). Therefore, it is essential to maintain an appropriate balance between the concentrations of these compounds. For example, increasing the concentration of calcium chloride (2%-3% w/v) decreased the encapsulation efficiency of protein hydrolysate extracted from *Ziziphus jujube* encapsulated due to saturation of the sodium alginate binding sites by calcium ions. Another aspect that affects encapsulation efficiency is the increased viscosity of the wall material solution (Kanbargi et al., 2017). This results in the formation of irregular and large droplets in which the formation of the polymer network and divalent ions is slower, causing the peptide to be released into the aqueous medium before encapsulation.

Ionic gelation is an efficient encapsulation technique to preserve the biological activity of bioactive peptides obtained from Ziziphus jujube seed (Kanbargi et al., 2017), rice husk (Ilhan-Ayisigi et al., 2021) and Soybean lupine (Pugliese et al., 2019). However, the *in vitro* release profile at neutral pH of rice husk peptides showed that after 6 days only 65% of the encapsulated peptides are released. This effect is due to the intermolecular interactions between the hydroxyl groups of the wall material and the peptide, as well as the strength of the gel formed (Ilhan-Ayisigi et al., 2021). An alternative to increase the release percentage of plant peptides can be the inclusion of low concentrations of wall materials that do not form gels in the presence of divalent ions and contribute to reducing gel strange, such as xanthan gum (Cano-Sampedro et al., 2020).

### 3.3 Liposomes

The encapsulation of bioactive peptides using liposomes involves the production of lipid bilayers similar to those found in the plasma membrane. Liposomes are vesicular structures composed of lipids arranged in bilayers or multilayers, formed from compounds capable of encapsulating hydrophilic and hydrophobic bioactive compounds such as cholesterol or phospholipids (Li et al., 2019). These lipid-based vesicles consist of one or more lipid bilayers, with the polar heads of the lipids facing both inwards and outwards. Because of their composition, they are a safe microencapsulation method for drug and bioactive compound delivery. Liposomes create barriers resistant to the enzymes in the mouth and gastrointestinal tract, as well as digestive juices, bile salts, and the intestinal flora. Hence, they protect bioactive compounds and improve their absorption and activity (Zhang et al., 2022).

Liposomes are categorized according to their size and the lipid bilayers in the membrane. Based on the membrane composition, they are classified into four types: unilamellar, oligolamellar, multilamellar, and multivesicular. Unilamellar liposomes are the most employed due to their ease of preparation and membrane characteristics. Oligolamellar liposomes present manufacturing difficulties due to their requirement for controlled processes. As such, they are infrequently used for bioactive compound encapsulation, while multivesicular liposomes have become widely popular for drug delivery purposes (Jesorka and Orwar, 2008). The size classification of liposomes divides them into three categories: small (15–500 nm), large (500–1,000 nm), and giant (more than 1 µm in length) (Bozzuto and Molinari, 2015).

There are various methods for forming liposomes. The most used methods include emulsion formation, lipid film dehydration, methods using micelle-forming detergents, and solvent injection methods. Liposome manufacturing methods typically begin with a dry film of lipid compounds, to which an aqueous solution of the bioactive compound is added. This results in the production of multilamellar vesicles with a high encapsulation efficiency. However, liposomes exhibit a large particle size distribution, which can affect the release rate (Jesorka and Orwar, 2008). The efficiency of liposomes as bioactive compound carriers depends on the nature of their components, physicochemical properties, surface charge, size, and particle size distribution (Bozzuto and Molinari, 2015).

Liposomes are efficient systems for encapsulating bioactive peptides due to the ability of peptides to integrate well with the liquid core. Nevertheless, the encapsulation efficiency is often low due to uncontrolled and spontaneous liposome formation driven by entropy. In addition, the peptides diffuse at a high velocity through the fluid membrane of the liposome, leading to the uncontrolled release of bioactive compounds due to their poor physical stability when stored under environmental conditions. Nanoliposomes are an alternative that can increase encapsulation efficiency, improve stability and functionality, and control the release rate of bioactive peptides, resulting in a significant increase in peptide bioavailability and an improved liposome pharmacokinetic profile (Li et al., 2017; Mazloomi et al., 2020).

Bioactive peptides derived from plant sources, such as peanuts (Gong et al., 2016; Li et al., 2019) and beans (Chay et al., 2015), have been stabilized through nanoliposome formation. The efficiency of bioactive peptide encapsulation by liposome formation is significantly lower than that obtained by other encapsulation techniques, because peptides obtained from beans are hydrophilic, which hinders their interaction with the phospholipids used as wall material, altering the structure of the lipid bilayer and causing the formation of pores (Chay et al., 2015). One of the strategies employed to increase encapsulation efficiency is the microfluidization used in liposome formation. The homogenization pressure applied in microfluidization increases the encapsulation efficiency and reduces the particle size distribution (Gong et al., 2016). In addition, other lipid compounds, such as 1-Palmitoyl-2-oleoyl-sn-glycero-3-phosphocholine (Rezaei et al., 2020) and 1, 2-dioleoyl-3-trimethylammonium propane (Mansourian et al., 2014), can be incorporated to reduce the permeability of the phospholipid membrane.

### 3.4 Emulsions

Lipid compounds are commonly used in the pharmaceutical and food industries to encapsulate bioactive peptides through emulsions (Ge et al., 2022). This method improves the digestibility and absorption of bioactive compounds under gastrointestinal conditions (Salvia-Trujillo et al., 2018). The emulsion is a system composed of two immiscible liquids that form two phases (Plazola-Jacinto et al., 2019). The material that forms the droplets of the emulsion is known as the dispersed, discontinuous, or internal phase, meaning that the compound surrounding the droplets is known as the continuous or external phase. Emulsions are classified into oil-in-water and water-in-oil, depending on the spatial distribution of the phases. Emulsions in which oil droplets are dispersed in an aqueous phase are called oil-in-water emulsions. By contrast, a water-in-oil emulsion is formed when water droplets are dispersed in a continuous oil phase (McClements, 2018; Pereyra-Castro et al., 2019).

The emulsion formation process involves creating droplets from the dispersed phase and reducing their size through homogenization. This process uses different low- and high-energy equipment, such as high-speed blenders, high-pressure valve homogenizers, colloid mills, and rotor-stators (McClements, 2018). The mechanical and shear forces created during the homogenization process through the rotor-stator cause the formation of small and stable droplets of the dispersed phase throughout the continuous phase. The homogenization process of an emulsion is one of the stages that most influence its stability. Generally, emulsions with a smaller particle size and narrow distribution are more stable to agglomeration and coalescence. In this sense, rotor-stators are one of the most widely used pieces of equipment in emulsion preparation (Pereyra-Castro et al., 2019; Plazola-Jacinto et al., 2019).

Emulsions are unstable systems since the immiscible phases that form them separate with time. Phase separation occurs due to various mechanisms, such as creaming, sedimentation, phase inversion, flocculation, and coalescence (Goodarzi and Zendehboudi, 2019). Creaming and sedimentation are phenomena caused by a difference in phase density. Creaming means moving droplets upward due to their lower density than the surrounding liquid. Meanwhile, sedimentation means that the droplets move downward due to their higher density than the surrounding liquid. Phase inversion is the mechanism in which an oil-in-water emulsion becomes water-in-oil and *vice versa* (Urbina-Villalba, 2009). Flocculation and coalescence result from the aggregation of two or more droplets. In flocculation, the droplets maintain their structure, while in coalescence two or more drops combine to form a larger drop. In addition to the use of equipment that allows the formation of small and stable micelles, stabilizers, which are generally amphiphilic compounds capable of reducing the surface tension of the continuous phase, are used to prepare emulsions. The stabilizers most employed in emulsion stabilization are Tween, lecithin, and glycerol (Ge et al., 2022).

Encapsulation uses different emulsion types to protect bioactive compounds. The selection of an emulsion type depends on the solubility of the bioactive compound. The formation of oil-in-water emulsions is the method employed when the bioactive compound is hydrophobic (Anal et al., 2019). However, if the bioactive compound is hydrophilic, double emulsion formation is the method of choice. A water-in-oil-in-water double emulsion is a complex system in which small water droplets with bioactive compounds are dispersed on a larger oil droplet. The first emulsion is scattered on the continuous aqueous phase (Muschiolik and Dickinson, 2017; Choi et al., 2020).

Encapsulation of bioactive peptides extracted from plant sources through the formation of emulsions presents low encapsulation efficiency values (Agrawal et al., 2021; Ying et al., 2021), as they are thermodynamically unstable systems. Because of this, stabilizing compounds such as sodium dodecyl sulfate, polyglycerol polyricinoleate, lecithin, and spam 60 have been used to improve emulsion stability (Ismail et al., 2020). Polyglycerol polyricinoleate was the stabilizer that allowed the most stable emulsions because this compound has a lower HLB value and a higher affinity for hydrophobic compounds than spam 60 and lecithin. The increase in the concentration of this stabilizer caused a decrease in the micelle size of the emulsion, increasing its stability (Ying et al., 2021).

Another alternative employed to increase peptide encapsulation efficiency is the elaboration of double emulsions. Different studies have shown the effect of microencapsulation by double emulsions of the bioactive peptides from plant proteins (Agrawal et al., 2021; Ying et al., 2021). In these systems, the bioactive peptides in an aqueous solution mixed with hydrophobic compounds, and the emulsion formed mixed with a solution of other wall materials such as polyvinyl alcohol solution, maltodextrin, and modified starch that stabilize the micelles formed in the first emulsion, increase the encapsulation efficiency. The elaboration of double emulsions of peptides obtained from soy protein (Ying et al., 2021) and pearl millet (Agrawal et al., 2021) allowed for preserving their stability and biological activity. However, the second emulsion significantly reduced peptide concentrations. These results represent a disadvantage for this encapsulation method because a larger quantity of microcapsules is required to observe the therapeutic effect.

Bioactive peptide delivery can be effectively achieved by encapsulating these compounds by double emulsions, as these systems can release them by hydration of the polymeric layer of the second emulsion, tested in the *in vitro* release of pearl millet peptides encapsulated by a double emulsion system, whose model corresponds to zero-order and Hixson Crowel kinetics (Agrawal et al., 2021). However, the efficacy of this process depends on the materials used to make the emulsion. In this sense, it is relevant to evaluate the use of stabilizing compounds from natural sources to improve the quality and efficacy of this encapsulation method.

Table 1 presents some characteristics of bioactive plant peptide capsules, the highest encapsulation efficiency values are obtained by spray drying and ionic gelation, as these encapsulation methods result in the formation of solid particles, whereas emulsions and liposomes are liquids. On the other hand, liposomes and emulsions have a smaller particle size, which favors their absorption and the release of peptides. Peptide encapsulation is a highly effective method for preserving peptide stability and biological activity, although the efficiency of this approach also varies considerably according to the wall materials employed.

**TABLE 1:** Effect of encapsulation method and wall material on encapsulation efficiency and particle size of plant protein-derived bioactive peptide capsules.

Encapsulation method	Bioactive peptides source	Biological activity	Wall material	WMC (w/v %)	EE (%)	Size	References
Spray drying	Flaxseed	Antioxidant	Maltodextrin	NR	NR	∼2–13 µm	Akbarbaglu et al. (2019)
	Bean *(Phaseolus lunatus)*	Hypoglycemic Antihypertensive	Maltodextrin Gum Arabic	∼12	82	3–7 µm	Cian et al. (2019)
	Rapeseed	Antihypertensive	Rapeseed protein isolate	8	84–99	5–12 µm	Wang et al. (2015)
Ionic gelation	Rice husk	Anticancer	Chitosan	0.05	89	180–257 nm	Ilhan-Ayisigi et al. (2021)
	Jujube *(Ziziphus jujube)*	Antioxidant	Sodium alginate	2–2.5	27–74	∼800 µm	Kanbargi et al. (2017)
	Soybean Lupine	Hypoglycemic	RADA16 peptides	1	NR	NR	Pugliese et al. (2019)
Liposomes	Peanut	Antihypertensive	Soybean phospholipid, cholesterol, deoxycholic acid sodium salt	4.4	∼45–65	79–301 nm	Gong et al. (2016)
	Peanut	Antihypertensive	Lecithin, Cholesterol	0.4–2	67–74	47 nm	Li et al. (2019)
	Orange seed	Antioxidant	Lecithin, cholesterol, chitosan	0.1 0.2 0.4	80.61 86.09 84.45	138–850 nm	Mazloomi et al. (2020)
	Winged bean seeds	Antioxidant Antihypertensive	Soya lecithin	2	27.6	193 nm	Chay et al. (2015)
Emulsions	Pearl millet	Antioxidant	Polymer eudragit, polysorbate 20, polyvinyl alcohol solution	3 2	19–88	150–242 µm	Agrawal et al. (2021)
	Amaranth	Antihypertensive	Amaranth protein isolate, sunflower oil	1 2	NR	9 µm 11 µm	Suárez and Añón (2019)
	Soybean	Antihypertensive	Polyglycerol polyricinoleate, lecithin, oil, modified starch, maltodextrin	40 80	29–74	1–12 µm	Ying et al. (2021)

WMC: wall material concentration, EE: encapsulation efficiency, NR: not reported.

## 4 Wall materials

Encapsulation involves surrounding the bioactive compound with a wall material that protects it from damaging conditions that cause degradation (Chaudhary et al., 2021). The choice of wall material is critical in determining the encapsulation method, as its composition and structure directly impact on parameters such as encapsulation efficiency and controlled release. A wide range of wall materials are available for use in the encapsulation process. These include carbohydrates, proteins, and lipids (Peanparkdee et al., 2016). The compounds used as wall material in encapsulation must form a cohesive film around the bioactive peptides, stabilize them, and protect them from environmental factors that may cause their hydrolysis. They should also be inert, tasteless, and able to release bioactive peptides at specific locations and rates under control (Choudhury et al., 2021). Various wall materials, such as lipids, proteins, and polysaccharides, have been proposed for bioactive peptide encapsulation. Due to their structural stability and natural properties, polysaccharides are a promising choice for delivery materials (Thongcumsuk et al., 2023). The properties of the wall materials that are most used for the encapsulation of bioactive peptides obtained from plant proteins are described in more detail below.

### 4.1 Maltodextrin

Maltodextrin is a compound frequently used as a wall material in bioactive compound encapsulation due to its high solubility in water (Xiao et al., 2022). It is a polysaccharide typically derived from the acidic or enzymatic hydrolysis of starch from various sources, such as maize, potato, rice, and wheat. Due to its origin, it is formed by a mixture of long chains of high and low-molecular-weight D-glucose linked by α-(1, 4) and α-(1, 6) glycosidic bonds. It also contains low concentrations of glucose (2%–3%) and maltose (5%–7%), which give it a slightly sweet taste (Avaltroni et al., 2004; Li et al., 2023). Dextrose equivalents (DE) express the degree of maltodextrin hydrolysis. DE is an inverse value of the average degree of polymerization of the dehydrated glucose units. Maltodextrins with a DE range of 3–20 are used as a wall material for bioactive compound encapsulation. In an aqueous solution, maltodextrin forms helical chains that are arranged into a continuous and elastic network. The hydrophobic groups are oriented inward within the network. This network allows for the interaction of the polysaccharide with bioactive compounds, facilitating their encapsulation (Castro et al., 2016; Xiao et al., 2022).

DE maltodextrins indicate the glucose concentration in this polymer. Low values indicate a low glucose concentration and a high polysaccharide concentration. DE has an impact on the physicochemical properties of maltodextrin. With an increase in the degree of hydrolysis, there is an increase in the number of exposed hydrophilic groups within the molecule, which leads to more frequent interactions with water, increasing the solubility and hygroscopicity of maltodextrin (Nurhadi et al., 2016). This results in an increased intermolecular free volume, which reduces the solution viscosity and increases molecular mobility (Xiao et al., 2022).

More hydrolysis in maltodextrin provides better protection for the encapsulated bioactive compounds. This effect is due to the formation of particles with more regular surfaces and fewer vacuoles (Negrão-Murakami et al., 2017). A higher DE may increase molecular mobility, facilitate water migration during capsule dehydration, and reduce vacuole formation and surface irregularities. However, this increased mobility may also lead to the migration of hydrophilic bioactive compounds to the capsule surface, decrease the mechanical strength of capsules, and favor agglomerate formation. Therefore, when selecting the DE of maltodextrin, it is important to consider the characteristics of the bioactive compounds (Barthold et al., 2019).

Maltodextrin was employed as a wall material to encapsulate bioactive peptides from plant sources, specifically flaxseed (Akbarbaglu et al., 2019). Increasing the maltodextrin ratio enhanced the performance of the encapsulation process and decreased the moisture content, water activity, and hygroscopicity of the capsules, due to the hydrogen bonds formed between the amino groups of the protein hydrolysate and the OH groups present in the maltodextrin, causing improvement in its stability (Akbarbaglu et al., 2019; Sarabandi et al., 2019). However, this wall material permits high molecular mobility of peptides, causing their fast delivery. Accordingly, it is recommended that alternative materials be utilized to optimize the encapsulation efficacy (Li et al., 2017).

### 4.2 Sodium alginate

Sodium alginate is a commonly used compound for encapsulating bioactive compounds and preserving the biological activity of certain bioactive peptides derived from plant proteins. This compound is a polymer present as a structural component of marine algae and some bacteria, formed by units of α-glucuronic acid and β-manuronic acid, linked by glycoside bonds (1–4). The polymer chains contain a random sequence of intercalated manuronic acid (M) and glucuronic acid (G) blocks (Hecht and Srebnik, 2016). Sodium alginate chains are generally rigid due to low rotation of the glycoside bond and electrostatic repulsion originating from the charge on the forming groups. The rigidity of the polymer depends on its composition; chains with two blocks of glucuronic acid (GG) are more rigid than those with two blocks of manuronic acid (MM). The least rigid chains are those formed by the union of a manuronic acid unit and a glucuronic acid (MG) unit (Ching et al., 2015).

The composition of alginate plays a crucial role in its physical and mechanical properties and depends on the block sequence of chains. Consequently, the blocks create binding sites for the union of divalent ions, with G-blocks showing a greater affinity for divalent ions (Sánchez-Ballester et al., 2021). G-block chains cause the formation of diamond-shaped electronegative cavities into which divalent ions bind via the oxygen atoms in carboxylic acid groups. Four carboxylic acid groups of the G-block interact with a divalent ion to form this configuration, resulting in a three-dimensional structure known as an egg crate network (Ching et al., 2015). The gel strength is higher in alginates with a high G-block content than in those with a high number of M-blocks. The above is because G-blocks have a higher affinity for divalent ions, forming a spatial conformation that favors ionic crosslinking (Nahar et al., 2017).

When sodium alginate is dripped into a divalent ion solution, the gelation process is external, occurring with the ion’s diffusion into the alginate, whereas, in internal gelation, the divalent ions are in the alginate solution and are released into the solution by a change in pH, causing gelation. External gelation is instantaneous and produces non-uniform cross-linking, while internal gelation generates uniform particles (Bidarra et al., 2014). The gelation capacity of sodium alginate in divalent ions also depends on the ion exchange coefficient between the metal ion and the sodium ion present in the alginate structure. The binding capacity of alginate with divalent ions follows the following order: lead, copper, cadmium, barium, strontium, calcium, cobalt, nickel, zinc, and manganese. Although calcium has a lower ion exchange coefficient than lead, copper, or cadmium, it is the most used due to its non-toxicity. The affinity of alginate for divalent ions depends on its composition. Therefore, there is a difference in the ion exchange coefficient between alginates derived from different sources (Chaturvedi et al., 2019).

Like the affinity of alginate for divalent ions, the physicochemical properties of alginate also depend on its composition. In general, they are water-soluble compounds. However, their water solubility depends on the pH and the concentration of water-divalent ions. Intramolecular hydrogen bonds are formed when the solvent pH is lower than 3.5, causing gel formation. On the other hand, if the concentration of divalent ions in the solvent is high, alginate gelling can occur, decreasing its solubility. Aqueous solutions of sodium alginate behave as pseudo-plastic fluids. The viscosity of these solutions varies between 20 and 400 mPa.S, increasing as the concentration of sodium alginate in the solution increases (Hecht and Srebnik, 2016). Sodium alginate is a highly efficient compound for administering drugs and bioactive compounds due to its stability under upper gastrointestinal tract conditions (Wu et al., 2023).

The encapsulation efficiency, stability, bioavailability, and transcellular permeability of collagen bioactive peptides were improved by sodium alginate use as a wall material (Wu et al., 2023). Additionally, this wall material enables the controlled release of its contents (Thongcumsuk et al., 2023). These findings indicate the potential of this wall material to enhance the absorption of peptides, while protecting them from inactivation in the gastrointestinal tract.

### 4.3 Gum Arabic

Gum Arabic is a tree gum exudate produced mainly by the tree species *Acacia senegal* and *Acacia seyal*. The trees producing this gum grow mainly on the African continent, from Senegal to Somalia, and in northern Ecuador. The physicochemical properties of gum Arabic depend on the tree species from which it was obtained, as well as the climate and season of the year in which it was produced. However, it is generally a highly water-soluble compound (Williams and Phillips, 2009).

Gum Arabic is a complex polysaccharide that contains approximately 2% nitrogenous material. The polypeptide chain of this protein fraction consists of 250 amino acids, including mainly hydroxyproline, aspartic acid, serine, proline, threonine, and leucine, which are linked to the carbohydrate building blocks of gum Arabic by the amino acids serine and hydroxyproline. It is a highly branched molecule with a random globular spiral-like structure, consisting of a core of galactose units linked by β1-3 glycoside bonds, with branches of galactose linked by β1-3 and β1-6 bonds, as well as arabinose linked by β1-3 bonds. Rhamnose and glucuronic acid are placed at the molecule’s periphery where the branches end (Chranioti and Tzia, 2014). The structural arrangement of the gum generates compact molecules with low viscosity, which allows the use of high concentrations (30%) of this compound. The solution’s viscosity increases over time and decreases at high concentrations of electrolytes or hydronium ions. This phenomenon is due to the dissociation of the carboxyl groups (Mothé and Rao, 2000; Jafari et al., 2008).

Gum Arabic, when used as a wall material in bioactive peptide encapsulation, improves encapsulation efficiency. According to Cian et al. (2019), increasing the amount of gum Arabic in the wall material mixture used in *Phaseolus lunatus* peptide encapsulation significantly improved the encapsulation efficiency. These findings could be attributed to the ability of gum Arabic to generate films that stabilize peptides in the polymeric structure, minimizing their movement toward the surface of the capsule.

### 4.4 Chitosan

Chitosan is a cationic polysaccharide derived from the enzymatic deacetylation of chitin (found in the exoskeletons of insects and shrimp) by hydrolysis of the acetylamino groups in a strong alkaline medium at high temperatures. Chitosan is composed of D-glucosamine and N-acetyl-D-glucosamine units via β (1→4) O-glycosidic bonds (Primo et al., 2024). The structure of chitosan allows its use in edible film formulations, wound healing, and microencapsulation of bioactive compounds since it has properties such as low toxicity, hydrophobicity, easy degradability, biocompatibility, and antimicrobial activity (Tavares and Zapata Noreña, 2019).

The bioactive peptides of rice husk-derived protein hydrolysates encapsulated using chitosan maintained the cytotoxic effect and made them suitable candidates for pharmaceutical applications. However, the delivery was not complete (Ilhan-Ayisigiet al., 2021). This wall material is often mixed with other compounds like sodium alginate (Zheng et al., 2023), caffeic acid (Xun et al., 2023), and linseed gum (Zheng et al., 2023). These compounds improved the thermal stability and antioxidant properties of the microcapsules. Moreover, during *in vitro* gastrointestinal digestion, the microcapsules showed good acid resistance and intestinal release.

### 4.5 Whey protein

Whey protein is a by-product of the dairy industry that contains different globular proteins (Kalajahi et al., 2023). It exhibits beneficial nutritional, functional, and structural characteristics. Whey proteins come in two main forms: whey protein isolate (WPI), which has >90% protein, and whey protein concentrate (WPC), which has 30%–85% protein (Jiang et al., 2018; Price, 2018). Jiang et al. (2018) conducted a study to compare the physicochemical and emulsifying properties of WPI and WPC. After a 30-min heating process at 80, 85, and 90°C, the authors concluded that the WPI network was more stable, homogeneous, and dense compared to that of WPC. WPI generally consists of α-lactalbumin (α-LA), β-lactoglobulin (β-LG), bovine serum albumin (BSA), and trace amounts of immunoglobulins and other proteins (Jiang et al., 2018; Ferraz et al., 2022). The structure, amphiphilic, biological, and emulsifying activity of WPI has allowed it to be used as a natural material for coating systems and to improve the bioavailability of active ingredients. Furthermore, concentration in high proportions allows the masking of flavors and results in color loss (Ma et al., 2014; Khalifa et al., 2019; Ferraz et al., 2022).

Whey proteins are suitable for creating microcapsules and gels without resorting to enhanced heat treatments or other substances due to their suitable film-forming, emulsification, solubility, and water-holding properties (Wang and Selomulya, 2020; Lekshmi et al., 2021). Due to its antipepsinic digestion, whey protein is an ideal wall material to encapsulate and release bioactive peptides, as the peptides are intended to resist degradation in the digestive environment of the stomach and reach intestinal absorption (Zhao et al., 2022). Ma et al. (2014) obtained whey protein concentrate hydrolysate (WPCH) and microencapsulated it to reduce its bitter taste and resistance to hygroscopicity without altering its immunoregulatory activity by spray drying and lyophilization using whey protein (WP) and sodium alginate (SA) as the wall material. The results showed that encapsulation only with whey protein without sodium alginate had higher solubility percentages due to the low solubility of sodium alginate. The authors concluded that the whey protein encapsulation process and spray drying help to decrease bitterness and hygroscopicity, and no adverse effects on the immunomodulatory activity of the whey protein hydrolysate were found.

Table 2 shows the values of some of the properties to be considered in the choice of wall material. Solubility is a property on which the intermolecular interactions between the wall materials and the peptide depend. As the solubility of the wall material increases, these interactions increase, favoring peptide encapsulation. Hygroscopicity favors the water absorption from the capsules and agglomerates formation and reduces the encapsulation efficiency. It is also necessary to consider viscosity, as solutions of greater viscosity generate particles of greater diameter. However, in these solutions, molecular mobility is reduced, which can be efficient in encapsulation. Materials with higher Tg are more stable because more energy is required to pass from the glassy to the rubbery state, which generates drastic changes in molecular mobility and stability of the capsules. Therefore, these properties could be considered in the design of capsules to regulate the peptide delivery.

**TABLE 2:** Properties of wall materials used on plant protein-derived bioactive peptide encapsulation.

Wall material	Solubility in water (%)	Viscosity (Pa.s)	Hygroscopicity (%)	Tg (°C)	References
Maltodextrin	97	10.22	20	178–184	Hadnađev et al. (2011), Khalifa et al. (2019)
Sodium alginate	76	0.683	21	229.9	Ma et al., 2014, Helmiyati and Aprilliza (2017), Chattopadhyay and Inamdar (2010)
Gum Arabic	80	1.19	25	133–189	Yebeyen et al. (2009), Khalifa et al. (2019)
Chitosan	NS	1.83	NR	222–239	Kasaai et al., 1999, Rao and Johns (2008)
Whey protein	79	13–15	13	44–53	Anker et al. (1999), Khalifa et al. (2019)

## 5 Controlled delivery

**FIGURE 4:**
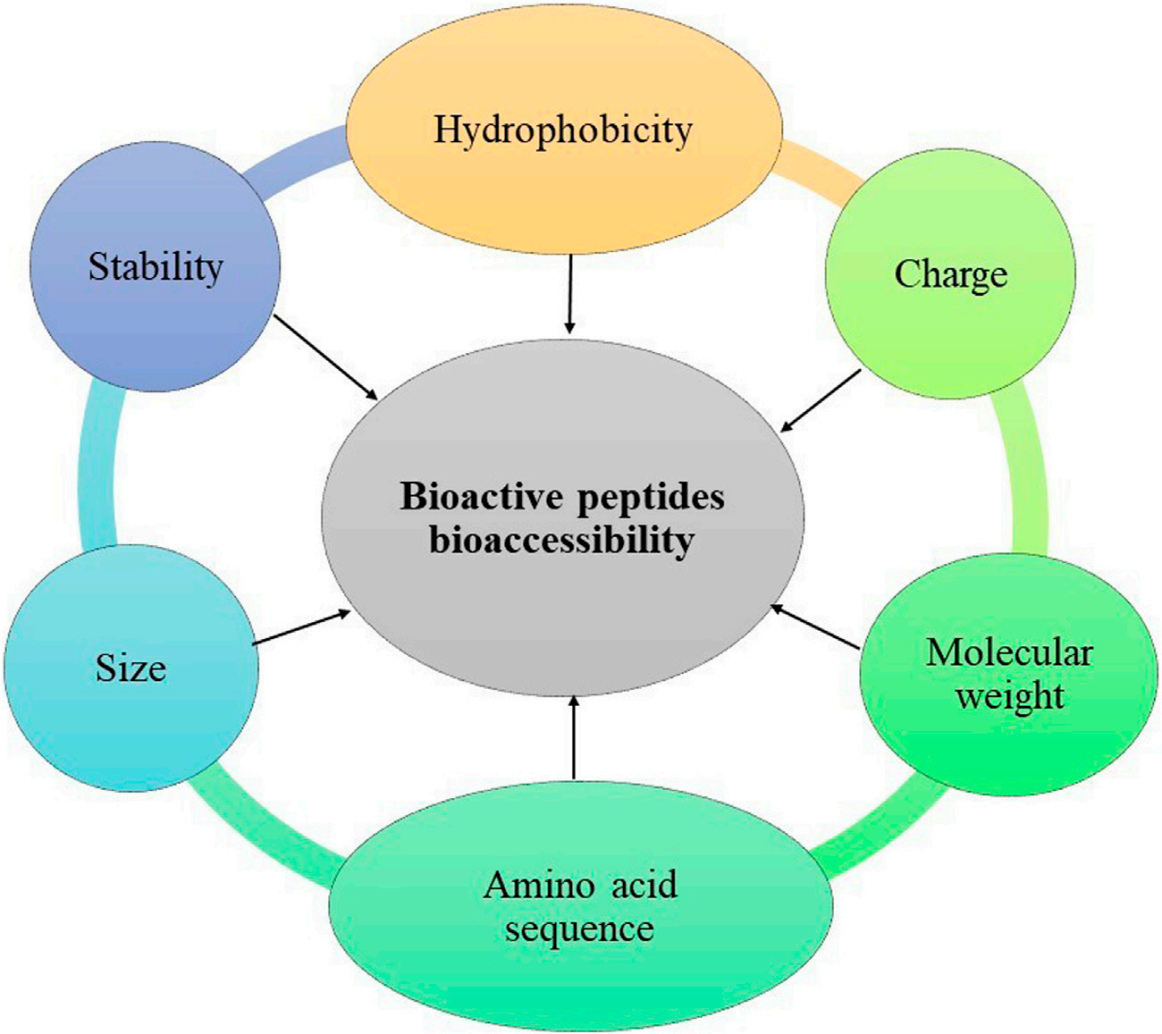
Plan-protein-derived peptide characteristics considered in the encapsulation method and wall material selection.

Controlled release is a mechanism by which the bioactive peptide is released under specific conditions at a predetermined rate and time (Martínez-Ballesta et al., 2018). Peptide release occurs primarily by diffusion. However, the diffusion rate can be modified by several mechanisms, such as changes in pore size, intermolecular interactions between the peptide and wall materials, and changes in pH or ionic strength. Other mechanisms by which peptides can be released from capsules include enzymatic activity and mechanical forces. Understanding the release mechanism of the bioactive peptide is crucial in controlling this process (Sobel et al., 2014; Bakry et al., 2016). To protect the biological activity of bioactive peptides, the delivery system must be able to prevent any change in their structure during encapsulation, storage, use of the final product, and administration. It must also enable the release of the peptides, making them bio-accessible and allowing them to be absorbed intact in the intestine for transportation to the target organs. Therefore, encapsulation methods and wall material selection must consider the nature of the peptide (Figure 4) (McClements, 2018; Udenigwe et al., 2021). The release of the peptide from the capsules and the rate at which this occurs depends on the composition and physicochemical characteristics of the capsule: size, shape, and particle microstructure (McClements, 2018).

Simple diffusion is the major mechanism of low-molecular-weight bioactive peptide release from capsules. This phenomenon depends on the concentration gradient, diffusion distance, and amount of matrix swelling (Kamaly et al., 2016). Simple diffusion occurs when the peptide size is smaller than that of the capsule pores. Therefore, capsule structure and pore size also affect the rate of peptide release. Increasing the size and number of pores in the capsule increases the rate of peptide migration (Aditya et al., 2017). The capsule size and shape also affect the delivery velocity. Smaller, spherical capsules have a larger contact surface, resulting in a faster release rate than larger, irregularly shaped capsules with a reduced contact surface. In small, spherical capsules, the peptides have a shorter distance to travel to the external environment, while the distance increases as the size increases (McClements, 2018).

Swelling causes an increase in pore size, and shrinkage results in diminished size. This phenomenon can occur due to changes in pH (James et al., 2014). When polymers with fillers are utilized as the wall material, they can change when interacting with the surrounding medium. This phenomenon is observed in capsules made of sodium alginate (negatively charged polymer) that tends to swell under neutral conditions and shrink under acidic pH conditions because the carboxylic groups of some chains of this compound lose their charge. Shrinkage of the capsules causes peptide retention, whereas swelling promotes their release (McClements, 2018).

Another factor that can cause the swelling or shrinkage of the capsules is the change in the ionic strength of the surrounding aqueous solution since it can modify the electrostatic repulsion between the polymer chains, causing their shrinkage (Quesada-Pérez et al., 2011). Chitosan is a cationic polymer, so adding salts, such as calcium or sodium chloride, causes its contraction due to electrostatic repulsion, decreasing the release rate of the peptides encapsulated with this wall material. A temperature change can also cause swelling or shrinkage of the capsules since the water absorption capacity of some wall materials increases when they are heated in an aqueous medium. These variables can be controlled to regulate the rate of peptide release by simple diffusion (De Kruif et al., 2015).

Intermolecular interactions between bioactive peptides and the polymers used for encapsulation hinder their release by simple diffusion. Linear polymers can conjugate to the termination of a peptide, destabilizing its three-dimensional structure. As the polymer length increases or the size of the peptide decreases, intermolecular interactions increase because as the size of the peptide decreases, its binding sites become less constrained, reducing agglomeration between the polymer monomers and increasing intermolecular interactions (Taylor and Jayaraman, 2020). Interactions between peptides obtained from plant sources and some polymers have been described previously. Corzo-Rios et al. (2018) evaluated the interactions between carboxymethylated Flamboyan gum and peptides obtained from *P. lunatus*. Results obtained by these authors showed that as the molecular size of the peptides decreased, the number of interactions with the gum increased. Therefore, intermolecular interactions should be considered to control the peptide release in wall material selection.

Peptides with a higher molecular weight than the capsule pore size are released by capsule dissociation. Erosion is a mechanism produced by interactions between capsules and the surrounding media in which the aqueous phase slowly penetrates through the polymeric matrix of the wall materials, causing their degradation, reducing capsule size, and increasing the peptide release rate. The enzymatic activity could also change the capsule structure, causing their dissociation; some polymers can be hydrolyzed or modified by enzymatic action (Devi et al., 2017). Modifying the polymer structure can lead to changes in the intermolecular interactions (hydrogen bridges, Van der Waals forces, electrostatic interactions) inside the capsule, causing its disintegration and, therefore, peptide release. Enzymes present in the digestive tract can hydrolyze some compounds used as wall materials, carbohydrates can be broken down by the action of amylases present in the mouth, some proteins are hydrolyzed by proteases in the stomach, and some other polymers can be hydrolyzed by enzymes secreted by bacteria in the large intestine (Zhang et al., 2016).

The bioavailability of bioactive peptides depends on their release in the large intestine, where enterocytes absorb them to enter the bloodstream and reach the target organs. Therefore, evaluating the release kinetics and biostability of encapsulated peptides during the digestive process is crucial to ensure their functionality at the target site. In this regard, special care must also be taken when selecting the method and wall materials used to encapsulate bioactive peptides to ensure their biostability and bioavailability.

## 6 Trends and opportunities

As described above, the main objective of bioactive peptide encapsulation is to protect their biological activity during ingestion and digestion, and to facilitate their release in target organs, improving their bioavailability. To achieve this depends on the encapsulation method and the wall materials employed. Therefore, research trends suggest the use of new methods and materials to increase the encapsulation efficiency in protecting peptide bioactivity.

The most common techniques for the encapsulation of peptides obtained from plant sources are liposomes, emulsions, and spray drying. However, liposomes and emulsions, being liquid systems, have low stability, while spray drying produces capsules with heterogeneous particle sizes and irregular shapes. These characteristics affect peptide biostability and bioavailability during the digestion process. In this context, using methods such as ionic gelation and coacervation represents a viable alternative for peptide encapsulation.

**FIGURE 5:**
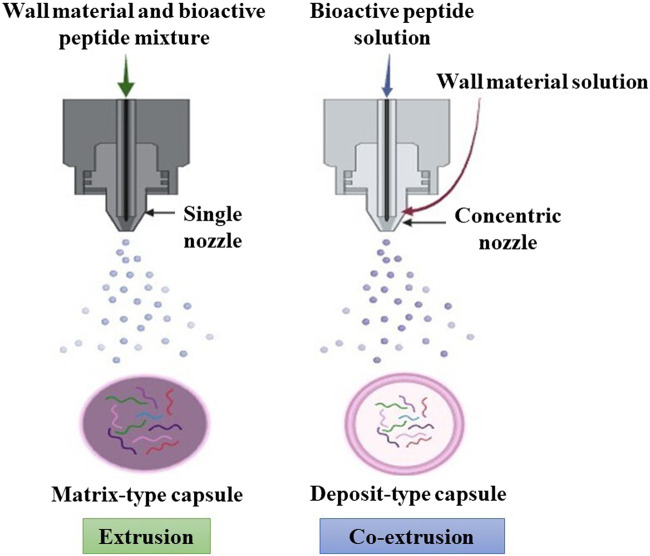
Plan-protein-derived peptide encapsulation process by extrusion and co-extrusion methods.

Various techniques, such as extrusion and co-extrusion, are available for the ionic gelation and coacervation (Bosnea et al., 2014; Timilsena et al., 2019) encapsulation of bioactive compounds (Demont et al., 2016; Kurozawa and Hubinger, 2017). Both methods can be used to produce capsules with uniform dimensions. However, the difference between them is that in extrusion, the wall material and the bioactive compound form a mixture that passes through the nozzle to form particles in which the bioactive compound is distributed within the matrix of the wall material. In co-extrusion, the bioactive compound passes through an internal nozzle while the wall material passes through an external nozzle to form capsules with a specific core (Figure 5) (Soliman et al., 2013; Demont et al., 2016).

The size, particle size distribution, and shape of the droplets produced by the above techniques depend on the viscosity and surface tension of the wall material, the nozzle size, and the pressure applied during droplet formation. However, as the process progresses, the spray surface expands, resulting in drops of different sizes. The different droplet sizes affect the velocity and distance the droplets travel, resulting in non-spherical particles of varying sizes. To avoid these problems, novel techniques incorporate a vibration system into the flow to improve the capsule size uniformity (Whelehan and Marison, 2011; Vemmer and Patel, 2013). Another modification to enhance the encapsulation process is electrostatic dispersion, in which a charge is applied to the surface of the droplets shortly after they form, inducing repulsion between them and preventing deformation. The charge application is useful for small particles, as larger droplets immerse into the divalent ion solution quickly enough to avoid distortion (Demont et al., 2016). Also, it is important to use as wall materials compounds that are not susceptible to the acid pH of the stomach to guarantee the release of the peptide in the intestine.

The molecular mobility and phase transitions of wall materials used in encapsulation determined the stability and release of bioactive peptides, especially when amorphous particles form. In glassy systems, the entrapment of bioactive compounds is favored, and their mobility is reduced (Rezvankhah et al., 2020). On the other hand, rubbery systems increase molecular mobility, leading to increased diffusion rates and degradation reactions of bioactive compounds. Some studies show that a mixture of inter-mixable wall materials increases the viscosity and glass transition temperature while decreasing molecular mobility. This effect increases the stability of the capsules, thus protecting the bioactive compound (Li et al., 2017).

Some compounds, such as gums and mucilage, have been employed as wall materials in the encapsulation of bioactive peptides. Among these compounds is Flamboyan gum (*Delonix regia*), which is a galactomannan formed by linear chains of D-mannopyranose (Man) linked by β1-4 bonds and branched D-galactopyranosyl (Gal) linked by α1-6 bonds. The ratio of Mal/Gal chains in this gum is 4:1 Mal/Gal (Corzo-Rios et al., 2014), like that of guar gum, but they differ in the position of the hydroxyl group in the main chain: in guar gum, it is linked by a beta bond and in Flamboyan gum, by an alpha bond. Due to the lack of electric charge in its chains, this wall material is not very soluble in water. Moreover, it presents a high viscosity, and these characteristics limit its application in the encapsulation of bioactive compounds as a wall material. However, carboxymethylation of uncharged gums represents a useful alternative to improve their physicochemical properties (Pacheco-Aguirre et al., 2010). Another alternative to increase the efficiency of Flamboyan gum as a wall material is to mix it with other compounds such as legume proteins since the interaction between these biomolecules is favored when the proteins are of low molecular weight (Corzo-Rios et al., 2018), which improves the rheological and textural properties of the mixtures (Corzo-Rios et al., 2014).

In addition to gums, chia mucilage (*Salvia hispanica* L.) has been utilized as a wall material in bioactive peptide encapsulation. Chia mucilage is a high-molecular-weight polysaccharide composed of polyuronides (xylose, glucose, and glucuronic acid units) that can form colloidal dispersions in water, increasing viscosity and with divalent cations are suitable for encapsulating drugs and bioactive compounds (Capitani et al., 2015). Chia mucilage contains fiber and protein, contributing to gel and capsule formation. Additionally, it improves the emulsification and water retention capacity of the wall material mixture (Waghmare et al., 2022). Urbizo-Reyes et al. (2019) utilized chia mucilage to form films with protein hydrolysates from the same seed, preserving their biological activity. The resulting films suggest that mucilage could serve as a wall material in bioactive peptide encapsulation.

The efficiency of the extrusion coupled to a vibration system in the encapsulation of soybean peptides was demonstrated by Cano-Sampedro et al. (2020) by obtaining high values of encapsulation efficiency (98%). Moreover, this technology allowed peptide concentration in the capsules without affecting the encapsulation efficiency or the spherical capsule shape. Sodium alginate and gum Arabic used as wall materials permitted the maintenance of the bead structure during the gastrointestinal digestion process, with a low percentage of protein hydrolysate released under these conditions. Therefore, these beads are a promising system for the delivery of bioactive compounds in the colon. The differences in the fermentation of the wall materials could be used as a regulator of the delivery rate of the compound.

The impact of encapsulation on the biological activity of peptides remains under-researched *in vivo* models. Hassan et al. (2021) evaluated the antimicrobial activity of mastoparan (a peptide derived from bees) encapsulated with chitosan by ionic gelation. The peptide demonstrated antimicrobial activity when administered to mice in encapsulated form. Chitosan protects the peptide from hydrolysis by proteases and peptidases, causes a synergistic bactericidal effect by damaging the integrity of the bacterial cell surface to a greater extent than non-encapsulated mastoparan. The delivery system produced good clinical results against drug-resistant strains of *A. baumannii. Nevertheless,* more studies evaluate the impact of encapsulation plan-protein-derived peptides on the *in vivo* models.

## 7 Conclusion

The review article demonstrates that encapsulating peptides bioactive derived from plant protein is a promising alternative to improve their stability, bioactivity, and bioaccessibility, enhancing their biological effects. The stability and bioaccessibility of encapsulated peptides increase when small, spherical particles with narrow-size particle distribution are obtained. Sodium alginate, due to the structure it forms, is the best material for protecting peptides from external factors such as light, temperature, pH, and enzymatic activity, thus increasing their stability during storage. However, the strong molecular structure formed by alginate makes it difficult for the peptides to be released and absorbed, reducing their bioaccessibility. This can be addressed by adding small amounts of other gums or mucilage to the wall material mix. Extrusion and co-extrusion are methods that allow sodium alginate to be used in combination with gums and mucilage as wall material to obtain spherical capsules with a narrow distribution of particle sizes. Furthermore, these methods enable the production of capsules with a higher peptide concentration without compromising encapsulation efficiency.
